# In This Issue

**Published:** 1997

## THE NEUROBIOLOGY OF ADDICTION

A person’s initial decision to use alcohol or other drugs (AOD’s) is influenced by genetic and environmental factors. Once ingested, however, the drug itself can encourage its continued use through direct action on nerve cells in the brain. According to Drs. Amanda J. Roberts and George F. Koob, the processes that lead to drug-seeking behavior and addiction result partly from altered communication among nerve cells and partly from activation of the brain regions involved in the body’s response to pleasurable stimuli. The authors postulate that a residual state of craving persists after stopping AOD use, which may prompt relapse, even in long-term abstainers. Research is beginning to reveal how specific brain regions may be integrated to form neural circuits that modulate aspects of addiction. This knowledge will aid in the development of improved treatment therapies. (pp. 101–106)

## NEUROTRANSMITTER REVIEW

Communication among brain cells plays a pivotal role in controlling the body’s functions, including movement, learning and memory, and thought. Accordingly, alcohol-induced disruption of this communication is the basis for many of alcohol’s effects on the body, particularly the brain. Nerve-cell communication is mediated by chemicals that excite or inhibit the impulse-receiving nerve cells (i.e., neurotransmitters) or modify the effects that neurotransmitters have on the impulse–receiving cells (i.e., neuromodulators). The articles in this special section review the functions and interactions of several neurotransmitter and neuromodulator systems. The authors also summarize current findings about alcohol’s effects on these systems, which may contribute to phenomena such as tolerance to and dependence on alcohol.

Dr. Gaetano Di Chiara describes the distribution and function of the neurotransmitter dopamine in the brain. Through its actions on the brain’s “reward” center, dopamine may contribute to motivation and reinforcement of alcohol consumption. (pp. 108–114)

Another neurotransmitter affected by alcohol is serotonin. According to Dr. David M. Lovinger, serotonin levels appear to be lower in the brains of alcoholics than in the brains of nonalcoholics. Serotonin also may promote alcohol’s intoxicating and rewarding effects by interacting with other neurotransmitter systems (e.g., dopamine). (pp. 114–120)

The brain’s major excitatory neurotransmitter is glutamate. Alcohol inhibits some of the receptors that mediate glutamate’s actions, state Dr. Rueben A. Gonzales and Mr. Jason N. Jaworski. These effects may account, in part, for the cognitive dysfunction associated with alcoholism. (pp. 120–127)

Drs. S. John Mihic and R. Adron Harris review alcohol’s effects on gamma-aminobutyric acid (GABA), the primary inhibitory neurotransmitter, and its main receptor, the GABA_A_ receptor. Enhanced GABA_A_-receptor function may be responsible for alcohol’s sedative effects. Moreover, alcohol-induced changes in GABA_A_-receptor function may contribute to alcohol dependence and tolerance, and abnormalities in the GABA system may contribute to the predisposition to alcoholism. (pp. 127–131)

Among the neuromodulators affected by alcohol are endogenous opioid peptides. These chemicals may play a significant role in mediating alcohol reinforcement and excessive alcohol consumption, writes Dr. Janice C. Froehlich. Consequently, medications that interfere with opioid-peptide functioning, such as naltrexone, can be important components of alcoholism treatment. (pp. 132–136)

Another neuromodulator altered by alcohol is adenosine. Drs. Douglas P. Dohrman, Ivan Diamond, and Adrienne S. Gordon summarize alcohol’s effects on the adenosine system and present evidence that adenosine mediates many of alcohol’s acute and chronic effects on the central nervous system, such as incoordination, intoxication, and sedation. (pp. 136–143)

## ALCOHOL AND NEUROTRANSMITTER INTERACTIONS

Alcohol acts on the brain by altering the function of neurotransmitters, chemicals that convey information from one nerve cell to another. However, the function of individual neurotransmitters cannot provide the entire explanation for a complex behavior like alcoholism. According to Dr. C. Fernando Valenzuela, alcohol produces its effects by interfering with the integration of multiple neurotransmitter systems, upsetting the delicate balance between excitatory and inhibitory signals. For example, the brain reacts to long-term alcohol exposure by increasing nerve cell arousal to counter alcohol’s general inhibitory effect. When alcohol consumption is abruptly stopped, the now overexcited nervous system produces symptoms of withdrawal. Improved understanding of these mechanisms will be important in the development of medications to treat alcoholism. (pp. 144–148)

## EXPLORING ALCOHOL WITHDRAWAL

After having stopped drinking, an alcoholic may experience a number of unpleasant symptoms collectively called the alcohol withdrawal syndrome. Drs. Deborah A. Finn and John C. Crabbe describe withdrawal and discuss clinical and experimental research into the mechanisms of its development. Symptoms of withdrawal—such as tremors, anxiety, elevated blood pressure, and convulsions—suggest hyperactivity of the nervous system. Evidence suggests that this hyperactivity represents the brain’s adaptation to the chronic inhibitory effect of long-term alcohol consumption on the cells involved in cellular communication: The symptoms are revealed when the alcohol is removed. Brain chemicals associated with adaptation and withdrawal may serve as a target for medications development. The authors recommend early treatment of withdrawal episodes, because symptom severity may increase with each subsequent withdrawal. (pp. 149–156)

## IMPLICATIONS FOR TREATMENT

Recent advances in neuroscience research have influenced the development of promising new treatments for alcoholism. Drs. Ismene Petrakis and John Krystal discuss research on three types of medications that exert their effects by altering chemical communication among nerve cells. One such medication, naltrexone, helps decrease the rate of relapse to drinking when used as an adjunct to psychosocial treatment for alcohol dependence. Another medication, acamprosate, may reduce alcohol consumption, perhaps in part by diminishing withdrawal symptoms that occur when the patient stops drinking. Finally, medications that affect the brain chemical serotonin have not consistently been found to reduce alcohol consumption; however, they may help relieve depression and anxiety, psychiatric disorders that may co-occur with alcoholism. (pp. 157–160)

## IS BEHAVIORAL TOLERANCE LEARNED?

After consuming alcohol, some drinkers seem to possess a degree of control over their behavior, even appearing to act sober when they are not. Dr. Muriel Vogel-Sprott explores several factors that may enhance the development of resistance (i.e., tolerance) to alcohol’s impairing effects on behavior. Dr. Vogel-Sprott reviews studies suggesting that drinkers will act intoxicated or sober after drinking depending on which type of behavior they believe will result in more favorable consequences. Drinkers rewarded for displaying sober behavior appear to learn new behavioral strategies to develop tolerance and compensate for alcohol’s impairing effects. (pp. 161–168)

